# Interactions of Gastrointestinal Peptides: Ghrelin and Its Anorexigenic Antagonists

**DOI:** 10.1155/2010/817457

**Published:** 2010-01-06

**Authors:** Anna-Sophia Wisser, Piet Habbel, Bertram Wiedenmann, Burghard F. Klapp, Hubert Mönnikes, Peter Kobelt

**Affiliations:** ^1^Division Hepatology, Gastroenterology, and Endocrinology, Department of Medicine, Charité—Universitätsmedizin Berlin, Campus Virchow, 13353 Berlin, Germany; ^2^Department of Medicine, Institute of Neurogastroenterology, Martin-Luther-Hospital, 14193 Berlin, Germany; ^3^Division Psychosomatic Medicine and Psychotherapy, Department of Medicine, Charité—Universitätsmedizin Berlin, Campus Mitte, 10117 Berlin, Germany

## Abstract

Food intake behaviour and energy homeostasis are strongly regulated by a complex system of humoral factors and nerval structures constituting the *brain-gut-axis*. To date the only known peripherally produced and centrally acting peptide that stimulates food intake is ghrelin, which is mainly synthesized in the stomach. Recent data indicate that the orexigenic effect of ghrelin might be influenced by other gastrointestinal peptides such as cholecystokinin (CCK), bombesin, desacyl ghrelin, peptide YY (PYY), as well as glucagon-like peptide (GLP). Therefore, we will review on the interactions of ghrelin with several gastrointestinal factors known to be involved in appetite regulation in order to elucidate the interdependency of peripheral orexigenic and anorexigenic peptides in the control of appetite.

## 1. Introduction

According to the current state of knowledge, control of food intake behaviour and energy homeostasis particularly relies on the complex interactions between various humoral components indicating the actual metabolic state of the organism. As a well-established hypothesis in the context of appetite regulation, the glucostatic theory suggests an important role of metabolic substrates (e.g., blood glucose levels) for the regulation of food intake [1]. Also, the assumed modulation of food intake by signals reflecting upon energy storage [2] has been validated by the discovery of the adipose tissue hormone leptin [3]. 

**Figure 1:**
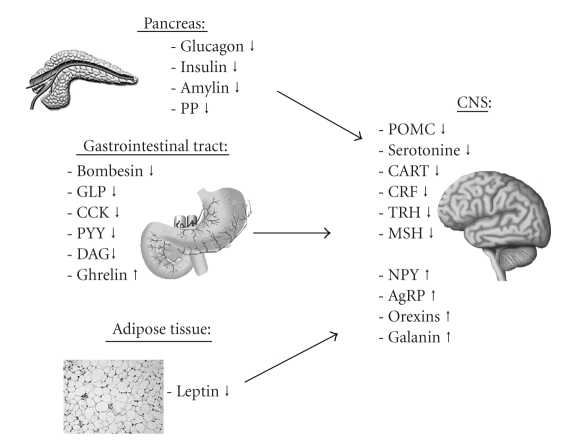
Peripheral and central peptides reducing (↓) or stimulating (↑) food intake (modified after Arora et al. [5]). AgRP: agouti-related peptide; CART: cocaine and amphetamine regulated transcript; CCK: cholecystokinin; CRF: corticotropin releasing factor; DAG: desacyl ghrelin; GLP: glucagon-like peptide; MSH: melanocyte stimulating hormone; NPY: neuropeptide Y; POMC: proopiomelanocortin; PP: pancreatic polypetide; PYY: peptide YY; TRH: thyreotropin releasing hormone.

During the past decades these theories were complemented by the discovery of several additional mechanisms involved in the control of energy homeostasis. Numerous studies revealed that diverse gastrointestinal peptides are particularly responsible for the control of hunger and satiety [4]. Serving as the most important gateway connecting the endocrine with the central nervous system (CNS), the hypothalamus has been found to comprise and integrate the humorally mediated information, which reflect the metabolic state of the organism [4]. This interaction between the central nervous system and the intestinal tract by humoral factors and neuronal pathways has been named *brain-gut-axis* [4]. As a part of the *brain-gut-axis* gastrointestinal neuropeptides as cholecystokinin (CCK), glucagon-like peptide-1 (GLP-1), peptide YY (PYY), and many other humoral components are mainly involved in short-term regulation of energy homeostasis. Figure 1 provides an overview of the sites of synthesis as well as of the effects exhibited by these peripheral and central peptidergic factors responsible for the regulation of hunger and satiety.

## 2. Ghrelin

So far the only known peripherally produced peptide exerting a stimulating effect on food intake behaviour is ghrelin [6]. In 1999 this peptide was discovered by Kojima et al. as the first endogenous ligand of the Growth Hormone Secretagogue Receptor (GHS-R) [6]. Ghrelin is a 28 amino acid peptide, which exhibits an esterification with an octanoyl chain at the serine residue on position three as an unique modification [6]. The acylation is catalyzed by the ghrelin-O-acyltransferase (GOAT) and converts the peptide to the biologically active form [7]. Moreover, the fatty acid residue has been found to be essential for the directed transfer *via* the blood-brain-barrier [8]. 

Ghrelin is mainly produced by mucosal X/A-cells of the stomach and in much smaller shares also in the pancreas, duodenum, small intestine, and coecum as well as in the heart and aorta [6, 9, 10]. Additionally, studies indicate that also regions of the brain are involved in the ghrelin synthesis as ghrelin-containing neurons were identified in the pituitary gland as well as in the arcuate nucleus of the hypothalamus [11, 12]. Moreover, ghrelin-immunopositive neurons have been described in a hypothalamic region located nearby the third ventricle [13]. 

Blood ghrelin levels rise preprandially, after weight loss and in the fasted state [14, 15]. Moreover, plasma ghrelin levels have been found elevated in mammals after H. pylori infection [16] as well as in patients suffering from peptic ulcers [17]. In addition, Masaoka et al. found an increase in plasma ghrelin levels and gastric preproghrelin mRNA expression in diabetic rats, whereas gastric ghrelin levels were decreased compared to nondiabetic animals [18]. In this context, zinc supplementation significantly reduced the density of ghrelin-producing cells in the fundic mucosa in diabetic animals in comparison to untreated nondiabetic controls [19]. 

In addition to a significant elevation of GH-secretion [6], exogenous ghrelin strongly stimulates food intake behaviour in rodents [14, 20–24] as well as in humans [25]. Likewise, elevated endogenous plasma levels of ghrelin in patients suffering from Prader-Willi-syndrome result in distinct hyperphagia [26]. In addition to its impact in the context of energy homoestasis ghrelin is also involved in the regulation of several intestinal functions, such as gastric acid secretion [27, 28] or extraintestinal actions, which are summerized in Table 1. 

Studies suggest that the orexigenic effect of ghrelin is mediated *via* central mechanisms located in the arcuate nucleus (ARC) of the hypothalamus. It has been shown that intracerebroventricular (icv.) injection of ghrelin leads to a significant increase of neuronal activity within ARC as well as in the paraventricular nucleus (PVN), dorsomedial nucleus of the hypothalamus (DMH), in lateral hypothalamic areas (LHA), in the nucleus of the solitary tract (NTS), and in the area postrema (AP) [29, 30]. Interestingly, intraperitoneal (ip.) injection of ghrelin has been found to induce neuronal activity in the ARC and PVN also, but yet failed to do so in the NTS and AP [31, 32]. However, after intravenous (iv.) ghrelin injection an increase in neuronal activity in the ARC, PVN, as well as in the NTS and AP [33] or activity within ARC, NTS, and AP but not in the PVN and DMH [34] has been reported.

Although the complete central mechanism of action remains to be elucidated, it is well established that the orexigenic effect of ghrelin is mediated *via* central pathways involving neuropeptide Y (NPY) and agouti-related peptide (AgRP) in the ARC [21, 35–39]. Accordingly, ghrelin does not effect food intake behaviour in NPY-/AgRP-deficient mice [38]. These findings and the colocalization of NPY and ghrelin receptor GHS-R1a in neurons of the ARC suggest that NPY- and AgRP-positive neurons are a basic prerequisite for the ghrelin-induced orexigenic effect [12, 36]. However, taking into account that the GHS-R1a is widely distributed in the brain [40], many other brain regions have been also found activated after ghrelin injection [32–34, 41, 42]. Therefore, it can be assumed, that there are further—yet unknown—mechanisms mediating the various effects of ghrelin. It is furthermore noteworthy that the effects of exogenous as well as endogenous ghrelin seem to be influenced by other factors of the brain-gut-axis. Therefore, some recent studies focused on the interaction between ghrelin and other humoral factors known to regulate hunger and satiety. These findings and their impact on the role of ghrelin in the hypothalamic system of food intake behaviour and energy homeostasis will be discussed in the following.

## 3. Interaction between Ghrelin and Peripheral Anorexigenic Peptides

### 3.1. Cholecystokinin

Cholecystokinin (CCK) was the first gut hormone found to reduce food intake [43]. CCK is secreted by I-cells located in the proximal small intestine as a mixture of peptides with varying numbers of amino acids, each of which possessing the required epitope for bioactivity [44]. It is widely accepted that CCK-induced satiation is mainly mediated by binding to CCK-1 receptors located on the vagus nerve [45, 46]. 

As the orexigenic effect of ghrelin is also partly mediated by vagal afferents, Date et al. found that peripheral injection of CCK curbs the decreased activity of gastric vagal afferents induced by ghrelin [23]. Besides, exogenous ghrelin significantly inhibits CCK-stimulated pancreatic protein secretion—even after acute subdiaphragmatic vagotomy [47]. Furthermore, it has been shown that elevated food intake after peripheral ghrelin administration is antagonized by pre- or simultaneous injection of CCK [48, 49]. Accordingly, the markedly increased neuronal activation of the hypothalamic ARC in response to peripheral ghrelin application is diminished by pre- or coapplication of CCK [48, 50]. However, peripheral ghrelin had no effect on CCK-induced neuronal activity in the PVN and the NTS [50]. Thus, it has been hypothesized that CCK inhibits the effect of ghrelin *via* vagal projections to hypothalamic pathways involving the ARC [50]. 

Interestingly, CCK-1 and -2 receptor deficient mice display a lower response to exogenous ghrelin and lower plasma ghrelin levels after fasting as compared to their wild-type littermates [51]. Moreover, intraduodenal infusion of ghrelin has been found to increase CCK secretion [52]. However, there are conflicting data concerning the influence of CCK on ghrelin release. Two studies indicated that exogenous CCK suppresses ghrelin release in healthy subjects, whereas after ingestion of lipids CCK seems to act on CCK-1 receptors to decrease ghrelin secretion [53, 54]. In contrast, it has been shown that CCK perfusion of isolated stomachs increases ghrelin secretion by ~200% [55]. 

In summary, there is good evidence for the functional antagonism of ghrelin and CCK on food intake whilst the exact interplay concerning the secretion of both peptides remains to be elucidated.

### 3.2. Bombesin

Bombesin is an anorexigenic tetradecapeptide initially isolated from the amphibian skin of Bombina bombina [68]. Since initial discovery, several mammalian bombesin-like peptides with structural homology to bombesin, such as gastrin-releasing peptide, neuromedin B, and neuromedin C, have been described [69]. Peripheral as well as central injection of bombesin reduces food intake mediated by bombesin receptors (BB1 and BB2) which are widely spread in the gastrointestinal tract as well as in the central nervous system [69–71]. Within the CNS, in particular the nucleus of the solitary tract of the brainstem has been shown to play a crucial role in the mediation of the anorexigenic effect of bombesin [72].

Concerning a possible interaction with ghrelin, evidence has been provided that coinjection of bombesin inhibits the orexigenic effect of intraperitoneal ghrelin [73]. In addition, simultaneous injection of bombesin and ghrelin significantly increased neuronal activity of CRF-immunoreactive neurons in the PVN compared to vehicle and to single ghrelin application while it did not alter ghrelin-induced neuronal activity in the ARC [73]. Therefore, it can be assumed that peripheral bombesin inhibits ghrelin-induced food intake and increases activation of CRF neurons in the PVN [73]. 

In addition, in goldfish (*Carassius auratus*) peripheral injection of bombesin diminished ghrelin expression levels in the gut [74]. Furthermore, while exhibiting opposing effects on food intake, application of exogenous bombesin and ghrelin both stimulated growth hormone release. However, the two peptides exerted different effects on somatostatin production, whereas peripheral ghrelin blocks the effects of bombesin on synthesis of the somatostatin mRNA [74]. Thus, the interactions between bombesin and ghrelin might account for postprandial variations found in serum GH levels and the forebrain expression of somatostatin mRNA [74]. 

In summary, bombesin directly interferes with sundry effects of ghrelin, most likely *via* central mechanisms.

### 3.3. Desacyl Ghrelin

The gastrointestinal peptide desacyl ghrelin (DAG) displays the identical amino acid sequence as ghrelin, however lacking the fatty acid residue [6]. Therefore, DAG—in contrast to ghrelin—does not interact with the GHS-R1a and thus was initially considered to be a degradation product of ghrelin without any biological effect [6]. However, recent literature indicates numerous actions of DAG (e.g., concerning cell proliferation and adipogenesis) [63, 64, 75–77]. In this context, it was found that transgenic mice over-expressing DAG showed a reduced food intake and a lower body weight compared to wild-type mice suggesting a role in the regulation of energy homeostasis [78, 79]. Also, exogenous DAG led to a significantly reduced cumulative body weight gain in adult male rats after one week of chronic infusion [80].

In addition, there is inconsistent data concerning a potentially anorexigenic effect of exogenous DAG [41, 79, 81, 82] that might be mediated by central pathways involving Urocortin and* Cocaine and Amphetamine Regulated Transcript *(CART) in the hypothalamic ARC and PVN [8, 41, 79]. However, data remain inconclusive.

Concerning a possible interaction between DAG and ghrelin, DAG was found to abrogate the metabolic effects of ghrelin after coadministration of both peptides [83]. More precisely, in rodents as well as in goldfish intraperitoneally administered ghrelin significantly increased food intake whereas simultaneously injected DAG abolished the stimulatory effect of ghrelin on feeding behaviour [83, 84]. Accordingly, the effect on neuronal activity in the ARC induced by ghrelin was significantly reduced when injected simultaneously with DAG [83]. As nesfatin-1 immunoreactive neurons in the ventromedial part of the ARC were activated by simultaneous injection of ghrelin and DAG, one might speculate that DAG suppresses ghrelin-induced food intake by curbing ghrelin-induced increased neuronal activity in the ARC and recruiting nesfatin-1 immunoreactive neurons [83].

Moreover, there is evidence indicating that DAG may counteract the role of ghrelin in the control of glucose metabolism. In humans exogenous ghrelin induced rapid changes in blood glucose and insulin levels, whereas DAG prevented the acylated ghrelin-induced effect when coadministered with acylated ghrelin [85, 86]. Furthermore, Gauna et al. found that glucose output by primary hepatocytes is time- and dose-dependently increased by incubation with ghrelin whilst this effect is counteracted by DAG coincubation [87]. Additionally, ghrelin-decreased insulin sensitivity has been reported to be prevented by intravenous coinjection of DAG [86, 88]. Besides interference with insulin secretion, in vitro DAG also abolished the effect of ghrelin on glucagon, pancreatic polypeptide, and somatostatin release [89].

Therefore, it can be summarized that DAG counteracts the effect of ghrelin on food intake, hypothalamic neuronal activation, glucagon, as well as on pancreatic polypeptide and somatostatin release. Furthermore, also opposing effects of DAG have been found on the effects of ghrelin covering insulin levels, sensitivity to insulin, as well as on blood glucose concentration.

### 3.4. Peptide YY

As a member of the pancreatic polypeptide family, peptide YY (PYY) is postprandially released from L-cells located in the distal gastrointestinal tract and has been reported to inhibit food intake *via* NPY-2 receptors expressed by neurons of the ARC [90, 91]. In addition to neurons of the ARC also vagal afferents projecting to the NTS have been found to be involved in the anorexigenic effect of PYY [92]. Based on the evidence that peripherally injected ghrelin acts *via* the N. vagus inducing neuronal activity in the ARC [24] a possible interaction of both peptides may be assumed theoretically. 

However, recent data are conflicting as one study showed PYY infusion to significantly reduces plasma ghrelin levels in humans [93] while other reports failed to find an influence on ghrelin concentrations in mice [94] and pigs [95]. Furthermore, in mice the anorexigenic effect of intraperitoneal PYY injection has not been found to be regulated by prevailing endogenous plasma ghrelin concentrations or coinjection of ghrelin [94]. However, in contrast Chelikani et al. reported peripheral ghrelin injections in rats to attenuate PYY-induced inhibition of food intake and gastric emptying [96]. In support of these results, Riediger et al. observed in rats that subcutaneous PYY directly inhibited ghrelin-activated neurons of the ARC [97].

Taken together, available data remain inconclusive concerning the interactions of ghrelin and PYY with a need for further investigation.

### 3.5. Glucagon-Like Peptide

The 31 amino acid hormone glucagon-like peptide (GLP) belongs to the incretins and is postprandially secreted by L-cells in the ileum [98, 99]. The peptide has been found to significantly reduce energy intake, gastric emptying rate, and energy consumption in humans [100]. 

In the context of interaction, it has been shown that icv. injection of GLP-1 significantly inhibited ghrelin-induced stimulation of food intake [101]. Vice versa, also intravenous coinfusion of ghrelin has been found to significantly attenuate the GLP-1-induced reduction of food intake and its inhibitory effect on gastric emptying [96].

Moreover, it is noteworthy that GLP-1 administration has been found to prevent the initial postprandial decline in ghrelin levels, possibly due to delayed gastric emptying [102]. Furthermore, exogenous GLP-1 significantly decreased ghrelin secretion after meal ingestion in healthy man [102] as well as during vagal prestimulation in isolated rat stomachs [103]. Also, application of “the closely related peptide” GLP-2 has been reported to reduce ghrelin concentrations in humans [104]. However, Brennan et al. observed that intravenous GLP-1 injection did not exhibit any effect on ghrelin concentrations in healthy humans [53].

In conclusion, there is some evidence that GLP might diminish ghrelin-triggered effects on food intake and gastric emptying and lead to a reduction of ghrelin release.

### 3.6. Amylin

Amylin is an anorexigenic peptide hormone composed of 37 amino acids, which is cosecreted with insulin from pancreatic islet *β*-cells in response to nutrient ingestion, incretin hormones, and neural input [105, 106]. Acute as well as chronic administration of amylin has been found to reduce food intake and body weight, which is predominantly mediated by neurons located in the area postrema [107, 108]. 

Initially, it has been shown that coadministration of amylin did not alter ghrelin-induced hyperphagia in rats [73]. In accordance, Osto et al. observed that the anorexigenic effect of amylin injection remained unchanged by simultanous ghrelin application in rats [109]. Thus it may be hypothesised that the metabolic state—*ad libitum* fed [73] or fasted [109] —of the animals might determine whether effects of ghrelin or amylin are predominant. 

However, in conclusion interaction between ghrelin and amylin seems to be unlikely.

### 3.7. Pancreatic Polypeptide

The 36 amino acid peptide pancreatic polypeptide (PP) is mainly produced by cells located in the periphery of endocrine pancreatic islets. Secretion of PP is stimulated postprandially and peripheral injection of PP in rodents as well as in humans has been shown to reduce food intake and body weight, most likely mediated *via* indirect effects on the hypothalamic ARC involving the area postrema [110, 111]. 

Arosio et al. reported that peripheral injection of ghrelin in humans leads to a significant increase of PP levels in healthy subjects but to have a variable effect on PP release in acromegalic patients [112, 113]. In contrast, Qader and colleagues observed a dose-dependent inhibitory effect of ghrelin perfusion on PP secretion of rodents' isolated islet cells [89].

Due to this conflicting data and the lack of studies investigating coinjection of both peptides the interplay between ghrelin and PP remains to be further elucidated.

### 3.8. Insulin

The 51 amino acid peptide insulin is produced by pancreatic beta islet cells and is commonly recognized as the most important hormone regulating glucose homeostasis. Central injection of insulin has been shown to reduce food intake as well as body weight [114], most likely mediated *via *insulin receptors expressed on ARC neurones [115]. High blood glucose levels increase insulin release and likewise ghrelin treatment in rats has been shown to stimulate insulin secretion from isolated pancreas tissue [116, 117] as well as in vivo [118]. In contrast, in experiments conducted by other investigators ghrelin perfusion of isolated rodents pancreas suppressed insulin release in response to glucose and other secretagogues [89, 119–121] and portal vein infusion of ghrelin inhibited the glucose-induced insulin secretion [122]. In line with these results, ghrelin administration decreased insulin serum levels in rats in vivo [59, 60, 123]. Accordingly, ghrelin infusion likewise significantly suppressed C-peptide levels in gastrectomized humans [124]. 

However, in growth hormone-deficient humans, peripheral ghrelin induced a rapid increase in plasma insulin levels, a stimulation of lipolysis, and a reduced peripheral insulin sensitivity [86, 125]. Interestingly, in ghrelin knockout mice the usually displayed high-fat diet-induced glucose intolerance was largely prevented [126] and also ghrelin receptor knockout mice were found to have an increased insulin sensitivity [127]. Also in ob/ob mice an improvement of the diabetic phenotype has been observed after the ablation of ghrelin [128].

Vice versa, most studies revealed an inhibitory effect of exogenous insulin on ghrelin levels in humans [129–132], rats [133, 134] as well as in isolated rat stomachs [55, 103, 135]. Moreover, Murdolo et al. observed that insulin seems to be essential for the prandial suppression of ghrelin levels in humans [136]. However, challenging these results Caixas et al. found that parenteral insulin does not influence blood levels of ghrelin in humans [137], while Toshinai and colleagues even observed increased ghrelin mRNA levels in the stomach after insulin administration [138].

Furthermore, during ghrelin infusion, insulin-dependent suppression of endogenous glucose production in mice has been reported to be less effective [88]. However, coadministration of ghrelin stimulated the insulin-induced glucose uptake in adipocytes [139]. Additionally, in hepatoma cells ghrelin has been identified to regulate downstream molecules of insulin signalling [140]. As antighrelin antibodies abolished the insulin-induced neuronal activation within the nucleus tractus solitarii of the brainstem, Solomon et al. concluded that this brain area might participate in peripheral ghrelin hunger signalling mediated by insulin [141].

Taken together, ghrelin and insulin obviously interfere in the reciprocal secretion regulation in a very complex manner.

## 4. Summary

Discovered in 1999, investigation of ghrelin as well as ghrelin-dependent effects and interactions is a quite novel field of research. However, during the last decade effects of ghrelin have been subject to intensive investigation. As obesity is a challenging problem worldwide, especially the orexigenic effect of ghrelin has been extensively explored. In this context, various possibilities to curb the stimulating effect on food intake behaviour have been investigated with more or less promising results [142, 143]. However, so far no substance has been identified to reliably inhibit food intake during long-term treatment. Nevertheless, it has been shown that the stimulatory effect of ghrelin on food intake is diminished by several anorexigenic peptides such as CCK, bombesin, desacyl ghrelin, PYY, insulin, and GLP but not by amylin. Some of these peptides inhibit ghrelin secretion and exert opposite effects on hypothalamic neuronal activity or gastric emptying. Thus, interaction between ghrelin and these anorexigenic gastrointestinal hormones might be an auspicious approach in the context of pharmacological obesity treatment.

Moreover, in addition to the previously introduced peptides originating from the gastrointestinal tract, also the satiety factor leptin, which is primarily synthesized in the adipose tissue, interacts with ghrelin. In this context, it has been described that leptin and ghrelin diminish each others' effects on food intake *via* oppositional influence on NPY-positive neurons within the ARC [37, 39]. Furthermore, as summarized in Table 2, both peptides interfere in various other ways [154, 155]. 

Taken together, during the last decade many aspects of appetite regulation associated with ghrelin have been elucidated. However, the brain-gut-axis—including ghrelin as the only peripheral orexigenic peptide—is a very complex system, for which our understanding to date remains limited. Thus, we can be curious for the next decades of ghrelin and its role in appetite regulation.

## Figures and Tables

**Table 1:** Physiological effects of ghrelin.

Reference	Physiological effect
Masuda 2000, Dornoville 2004 [56, 57]	Increased gastrointestinal motility
Masuda 2000, Date 2001 [56, 58]	Influence on gastric acid secretion
Broglio 2001, Dezaki 2004, Yada 2008 [59–61]	Reduction of insulin secretion
Nagaya 2001 [62]	Decreased blood pressure
Baldanzi 2002 [63]	Inhibition of apoptosis in cardiomyocytes
Cassoni 2001 [64]	Inhibition of proliferation in breast cancer
Weikel 2003 [65]	Extension of slow-wave sleep
Asakawa 2001, Carlini 2002 [66, 67]	Anxiogenesis and memory consolidation

**Table 2:** Interference between ghrelin and leptin.

Reference	Interaction
Barazzoni 2003 [144]	Leptin injection reduces starvation-induced ghrelin secretion.
Toshinai 2001 [138]	Leptin administration increases ghrelin mRNA level in the stomach
Dixit 2004 [145]	Ghrelin inhibits leptin-induced cytokine expression
Nakazato 2001, Kim 2004 [21, 146]	Ghrelin reverses leptin-induced feeding reduction
Shintani 2001, Kohno 2003, Kohno 2007 [37, 39, 147]	Leptin suppresses Ghrelin-induced activation of NPY neurons within the ARC
Rosicka 2003, Park 2005 [148, 149]	Ghrelin and leptin levels are reversely correlated and depend on the BMI
Bagnasco 2002, Beretta 2002, Dube 2002, Bagnasco 2003 [150–153]	Central transgenic leptin expression elevates serum ghrelin levels
